# Supplementary material for: An empirical evaluation of genotype imputation of ancient DNA
Source: G3 (Bethesda). 2022 Apr 28;12(6):jkac089. doi: 10.1093/g3journal/jkac089 (PMC9157144; doi:10.1093/g3journal/jkac089)
Supplement: jkac089_Supplementary_Data [file jkac089_supplementary_data.pdf]

Supplementary material for

An empirical evaluation of genotype imputation of ancient DNA

Kristiina Ausmees, Federico Sanchez-Quinto, Mattias Jakobsson, Carl Nettelblad

Data

Table S1: The high-coverage samples used for performance evaluation.

High-coverage samples			
	**Sample**	**Coverage (approx)**	**Reference**
1	Loschbour	22	Lazaridis et al. 2014
2	LBK	19	Lazaridis et al. 2014
3	ans17	27	Fraser and Sánchez-Quinto et al. (in preparation)
4	ne1	22	Gamba et al. 2014
5	sf12	57	Günther et al. 2018

Table S2: The low- to moderate-coverage samples included in the imputation panel in configurations 2 and 3.

Low- to moderate-coverage samples			
	**Sample**	**Coverage (approx)**	**Reference**
1	BA64	9.68	Cassidy et al. 2016
2	Gok2	1.19	Skoglund et al. 2014
3	LC41	0.95	Martiniano et al. 2017
4	LC42	2.60	Martiniano et al. 2017
5	LC44	1.77	Martiniano et al. 2017
6	CA122A	1.52	Martiniano et al. 2017
7	CM9B	2.56	Martiniano et al. 2017

8	DOLDA96B	1.69	Martiniano et al. 2017
9	CB13	0.85	Olalde et al. 2015
10	Iceman	4.44	Keller et al. 2012
11	ne5	0.71	Gamba et al. 2014
12	ne6	0.86	Gamba et al. 2014
13	ne7	0.83	Gamba et al. 2014
14	CO1	0.79	Gamba et al. 2014
15	Bar8	6.28	Hofmanová et al. 2016
16	Bar31	3.37	Hofmanová et al. 2016
17	ATP2	8.71	Günther et al. 2015
18	ATP16	12.98	Günther et al. 2015
19	ATP12	2.43	Günther et al. 2015
20	mur	3.33	Valdiosera et al. 2018
21	ans8	1.94	Sánchez-Quinto et al. 2019
22	ans14	2.58	Sánchez-Quinto et al. 2019
23	ajv70	1.34	Günther et al. 2018
24	ajv58	2.68	Günther et al. 2018
25	prs2	1.16	Sánchez-Quinto et al. 2019
26	prs9	1.89	Sánchez-Quinto et al. 2019
27	prs13	1.56	Sánchez-Quinto et al. 2019
28	prs16	1.78	Sánchez-Quinto et al. 2019
29	bal4	1.54	Sánchez-Quinto et al. 2019
30	kol6	1.48	Sánchez-Quinto et al. 2019
31	CO1CP	0.17	Mathieson et al. 2015
32	ne1CP	0.08	Mathieson et al. 2015
33	ne6CP	0.21	Mathieson et al. 2015
34	ne7CP	0.20	Mathieson et al. 2015
35	Motala12CP	0.30	Mathieson et al. 2015
36	BranaCP	NA	Mathieson et al. 2015
37	KO1CP	0.20	Mathieson et al. 2015
38	Kotias	15.40	Jones et al. 2015
39	Satsurblia	2.16	Jones et al. 2015
40	Motala12SG	1.94	Lazaridis et al. 2014
41	Bichon	13.52	Jones et al. 2015
42	KO1	0.94	Gamba et al. 2014
43	LaBrana	2.78	Olalde et al. 2014
44	Paliambela	1.22	Hofmanová et al. 2016
45	Kleitos	1.92	Hofmanová et al. 2016
46	Revenia	1.02	Hofmanová et al. 2016
47	LatH1	0.86	Jones et al. 2017
48	LatH2	2.70	Jones et al. 2017
49	LatH3	0.62	Jones et al. 2017
50	LatMN1	0.12	Jones et al. 2017

51	Canes1	0.87	González-Fortes et al. 2017
52	SC1	0.98	González-Fortes et al. 2017
53	SC2	2.70	González-Fortes et al. 2017
54	OC1	1.51	González-Fortes et al. 2017
55	H26	4.00	Günther et al. 2018
56	SF9	1.15	Günther et al. 2018
57	sbj	0.43	Günther et al. 2018
58	H22	0.71	Günther et al. 2018
59	steigen	1.24	Günther et al. 2018
60	Kunila2	0.31	Mittnik et al. 2018
61	Gyvakarai1	2.00	Mittnik et al. 2018

Table S3: The number of markers considered for performance evaluation for each high-coverage sample and coverage level. The first row specifies the number of markers at which the filtered HQ data overlaps with the loci used in the imputation; these are the sites for which genotype concordance can be calculated. Subsequent rows show, for each coverage level that the high-coverage data was downsampled to, how many of the total sites had overlapping reads in the low-coverage data, and how many did not.

**Number of markers used in performance evaluation**						
		**ans17**	**sf12**	**LBK**	**Loschbour**	**ne1**
total		26317153	27072696	15907150	17655877	19148120
0.1x	overlap	2064457	2054700	1437112	1516225	1516936
	no overlap	24252696	25017996	14470038	16139652	17631184
0.25x	overlap	4728775	4697958	3243931	3440090	3454067
	no overlap	21588378	22374738	12663219	14215787	15694053
0.5x	overlap	8223690	8159398	5535230	5888457	5974794
	no overlap	18093463	18913298	10371920	11767420	13173326
0.75x	overlap	10816510	10737175	7147381	7629381	7801581
	no overlap	15500643	16335521	8759769	10026496	11346539
1.0x	overlap	12738211	12661508	8282383	8871611	9136265
	no overlap	13578942	14411188	7624767	8784266	10011855
1.25x	overlap	14160480	14098034	9073516	9747395	10105258
	no overlap	12156673	12974662	6833634	7908482	9042862
1.5x	overlap	15225117	15186672	9625059	10370551	10801828
	no overlap	11092036	11886024	6282091	7285326	8346292
1.75x	overlap	16011058	15999422	10002613	10811681	11307364
	no overlap	10306095	11073274	5904537	6844196	7840756
2x	overlap	16600114	16620928	10262561	11120285	11666168
	no overlap	9717039	10451768	5644589	6535592	7481952

Results

Figure S1: PCA comparing HQ, imputed and low-coverage data for the five evaluation individuals. A reference PCA was defined based on genotypes of modern European samples from the Human Origins [**Patterson2012**] data set, after which the KDR method was used to estimate scores of ancient individuals. Modern individuals are indicated by gray dots and ancient individuals coloured according to the legend. The low-coverage data points correspond to the genotypes that have been downsampled to 1x, filtered, and used as input to imputation, which was performed using configuration 3 (Table 1 in the main article text), with a posterior filter of minimum genotype probability of 0.99 applied.

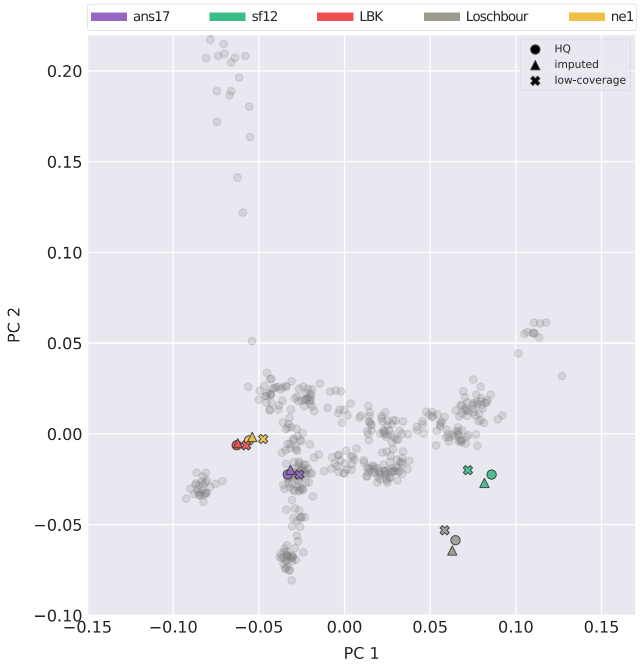

References

[1] Iosif Lazaridis et al. “Ancient human genomes suggest three ancestral populations for present-day Europeans”. In: *Nature* 513.7518 (Sept. 2014), pp. 409–413.

[2] Cristina Gamba et al. “Genome flux and stasis in a five millennium transect of European prehistory”. In: *Nature Communications* 5 (Oct. 2014), p. 5257.

[3] Torsten Günther et al. “Population genomics of Mesolithic Scandinavia: Investigating early postglacial migration routes and high-latitude adaptation”. In: *PLOS Biology* 16.1 (Jan. 2018), pp. 1–22.

[4] Lara M. Cassidy et al. “Neolithic and Bronze Age migration to Ireland and establishment of the insular Atlantic genome”. In: *Proceedings of the National Academy of Sciences* 113.2 (2016), pp. 368–373.

[5] Pontus Skoglund et al. “Genomic diversity and admixture differs for Stone-Age Scandinavian foragers and farmers”. In: *Science* 344.6185 (2014), pp. 747–750.

[6] Rui Martiniano et al. “The population genomics of archaeological transition in west Iberia: Investigation of ancient substructure using imputation and haplotype-based methods”. In: *PLOS Genetics* 13.7 (July 2017), pp. 1–24.

[7] Iñigo Olalde et al. “A common genetic origin for early farmers from Mediterranean Cardial and Central European LBK cultures”. In: *Molecular Biology and Evolution* 32.12 (Sept. 2015), pp. 3132–3142.

[8] Andreas Keller et al. “New insights into the Tyrolean Iceman’s origin and phenotype as inferred by whole-genome sequencing”. In: *Nature Communications* 3.1 (2012), p. 698.

[9] Zuzana Hofmanová et al. “Early farmers from across Europe directly descended from Neolithic Aegeans”. In: *Proceedings of the National Academy of Sciences of the United States of America* 113.25 (June 2016), pp. 6886–6891.

[10] Torsten Günther et al. “Ancient genomes link early farmers from Atapuerca in Spain to modern-day Basques”. In: *Proceedings of the National Academy of Sciences* 112.38 (2015), pp. 11917–11922.

[11] Cristina Valdiosera et al. “Four millennia of Iberian biomolecular prehistory illustrate the impact of prehistoric migrations at the far end of Eurasia”. In: *Proceedings of the National Academy of Sciences* 115.13 (2018), pp. 3428–3433.

[12] Federico Sánchez-Quinto et al. “Megalithic tombs in western and northern Neolithic Europe were linked to a kindred society”. In: *Proceedings of the National Academy of Sciences* 116.19 (2019), pp. 9469–9474.

[13] Iain Mathieson et al. "Genome-wide patterns of selection in 230 ancient Eurasians". In: *Nature* 528 (Nov. 2015), pp. 499–503.

[14] Eppie R. Jones et al. "Upper Palaeolithic genomes reveal deep roots of modern Eurasians". In: *Nature Communications* 6.1 (2015), p. 8912.

[15] Gloria González-Fortes et al. "Paleogenomic evidence for multi-generational mixing between Neolithic farmers and Mesolithic hunter-gatherers in the lower Danube basin". In: *Current Biology* 27.12 (June 2017), pp. 1801–1810.

[16] Alissa Mittnik et al. "The genetic prehistory of the Baltic Sea region". In: *Nature Communications* 9.1 (2018), p. 442.
